# Predictive Efficacy of a Radiomics Random Forest Model for Identifying Pathological Subtypes of Lung Adenocarcinoma Presenting as Ground-Glass Nodules

**DOI:** 10.3389/fonc.2022.872503

**Published:** 2022-05-12

**Authors:** Fen-hua Zhao, Hong-jie Fan, Kang-fei Shan, Long Zhou, Zhen-zhu Pang, Chun-long Fu, Ze-bin Yang, Mei-kang Wu, Ji-hong Sun, Xiao-ming Yang, Zhao-hui Huang

**Affiliations:** ^1^Department of Radiology, Affiliated Dongyang Hospital of Wenzhou Medical University, Dongyang, China; ^2^Department of Radiology, Sir Run Run Shaw Hospital, Zhejiang University School of Medicine, Hangzhou, China; ^3^Image-Guided Bio-Molecular Intervention Research, Department of Radiology, University of Washington School of Medicine, Seattle, WA, United States

**Keywords:** lung tumor, ground-glass nodules, radiomics, random forest, diagnosis

## Abstract

**Purpose:**

To establish and verify the ability of a radiomics prediction model to distinguish invasive adenocarcinoma (IAC) and minimal invasive adenocarcinoma (MIA) presenting as ground-glass nodules (GGNs).

**Methods:**

We retrospectively analyzed 118 lung GGN images and clinical data from 106 patients in our hospital from March 2016 to April 2019. All pathological classifications of lung GGN were confirmed as IAC or MIA by two pathologists. R language software (version 3.5.1) was used for the statistical analysis of the general clinical data. ITK-SNAP (version 3.6) and A.K. software (Analysis Kit, American GE Company) were used to manually outline the regions of interest of lung GGNs and collect three-dimensional radiomics features. Patients were randomly divided into training and verification groups (ratio, 7:3). Random forest combined with hyperparameter tuning was used for feature selection and prediction modeling. The receiver operating characteristic curve and the area under the curve (AUC) were used to evaluate model prediction efficacy. The calibration curve was used to evaluate the calibration effect.

**Results:**

There was no significant difference between IAC and MIA in terms of age, gender, smoking history, tumor history, and lung GGN location in both the training and verification groups (P>0.05). For each lung GGN, the collected data included 396 three-dimensional radiomics features in six categories. Based on the training cohort, nine optimal radiomics features in three categories were finally screened out, and a prediction model was established. We found that the training group had a high diagnostic efficacy [accuracy, sensitivity, specificity, and AUC of the training group were 0.89 (95%CI, 0.73 - 0.99), 0.98 (95%CI, 0.78 - 1.00), 0.81 (95%CI, 0.59 - 1.00), and 0.97 (95%CI, 0.92-1.00), respectively; those of the validation group were 0.80 (95%CI, 0.58 - 0.93), 0.82 (95%CI, 0.55 - 1.00), 0.78 (95%CI, 0.57 - 1.00), and 0.92 (95%CI, 0.83 - 1.00), respectively]. The model calibration curve showed good consistency between the predicted and actual probabilities.

**Conclusions:**

The radiomics prediction model established by combining random forest with hyperparameter tuning effectively distinguished IAC from MIA presenting as GGNs and represents a noninvasive, low-cost, rapid, and reproducible preoperative prediction method for clinical application.

## 1 Introduction

Ground-glass nodule (GGN) refers to a nodular shadow with slightly increased density on high-resolution computed tomography (HRCT), in which the vascular and bronchial bundles are not covered (1, 2). With the popularization of HRCT and the extensive application of low-dose screening for lung cancer, the detection rate of lung GGN has been constantly increasing (3). Lung GGN is a characteristic but non-specific imaging manifestation. Theoretically, with any decrease in air content in the lung tissue, increase in cell density, and proliferation of columnar cells in the alveolar wall leading to a decrease in gas filling in the terminal saccules and alveoli, ground-glass opacities can appear before alveoli collapse completely. Research has shown that persistent lung GGNs are mostly attributed to precancerous lesions or early-stage lung adenocarcinoma (4). The 2011 International Association for the Study of Lung Cancer/American Thoracic Society/European Respiratory Society International Multidisciplinary Lung Adenocarcinoma Classification (5) and WHO (2021) Classification of Lung Tumors Pathology (6) divided lung adenocarcinoma into three categories: pre-invasive lesions, minimally invasive adenocarcinoma (MIA), and invasive adenocarcinoma (IAC), among which pre-invasive lesions include atypical adenomatous hyperplasia (AAH) and adenocarcinoma *in situ* (AIS). AAH, AIS, MIA, and IAC are a dynamic process of continuous progression involving multiple genes, and AAH and AIS can gradually develop into MIA and IAC (7). According to the literature, when a lung GGN was completely removed and the margin was negative, the 5-year disease-free survival of AIS and MIA was 100% or close to 100% (8), the 10-year disease-specific survival (DSS) was 100% or 97.3%, and the 10-year DSS of IAC was 74.8% or 80.2%. Thus, the prognosis of IAC was significantly worse than that of MIA and AIS (9). The difference in prognosis determines the difference in clinical diagnosis and treatment schemes. Although it is still controversial, most researchers believe that scheduled follow-up or sublobar resection (wedge resection or segmental resection) is suitable for pre-invasive lesions and MIA, which can preserve more lung tissue as well as reduce the mortality and morbidity related to surgery, while lobectomy should be performed for IAC (10, 11). Therefore, accurate preoperative differentiation between IAC and MIA+ pre-invasive lesions, especially IAC and MIA, will assist in determining the appropriate surgical methods and the judgment of prognosis (12).

Radiomics is a newly emerging field and was first proposed by Dutch scholars Lambin et al. It refers to extracting a large volume of data that are hard to observe with the naked human eye from medical images such as B-mode ultrasonography, CT, magnetic resonance imaging, and positron emission tomography; radiomics uses a data characterization algorithm to transform the medical image data into minable feature space data with high resolution (13). Quantifying the heterogeneity of tumors using radiomics analysis software can help to obtain more information. Moreover, radiomics is not affected by the inherent limitations of the professional level or subjective analysis and traditional image interpretation; it can help us to effectively carry out pathological classification, treatment plan formulation, and treatment outcome and prognosis evaluation, among other tasks. It is widely known that heterogeneity is a recognized malignant feature of tumors, which is related to their adverse biological behavior. The heterogeneity of tumors is related to various gene subtypes, growth expression, and neovascularization and tumor microenvironment factors, which lead to local differences in the proliferation, metabolic activity, apoptosis, and blood supply among different tumors (14). At present, radiomics has been gradually applied in differentiating benign from malignant pulmonary nodules (15), evaluating treatment outcomes of lung cancer (16), and predicting the recurrence and metastasis of lung cancer (17), among other tasks. There are few studies on predicting the pathological subtypes of GGN lung adenocarcinoma, and most of them were used to differentiate MIA/IAC from pre-invasive lesions (AAH/AIS). Weng et al. (18) attempted to differentiate IAC from MIA by combining morphology with omics; however, no research has been reported on differentiation between IAC and MIA by pure omics labeling models. In this study, random forest (19) combined with hyperparameter tuning was used to establish and verify the ability of a radiomics prediction model to distinguish IAC and MIA presenting as lung GGN and to evaluate the consistency between the probability predicted by the model and the actual probability.

## 2 Materials and Methods

### 2.1 Research Subjects

The study design was approved by the appropriate ethics review board, and the requirement for obtaining informed patient consent was waived owing to the retrospective nature of the study.

In this study, we retrospectively analyzed data from patients treated in our hospital from March 2016 to April 2019. The inclusion criteria were as follows (6, 20, 21): (i) GGN on pre-operation chest HRCT scans; (ii) the images were scanned using the same scanning protocol on the same CT machine; (iii) presence of lesions on at least five sections on HRCT axial images; (iv) all lesions were confirmed as lung IAC or MIA by pathology after surgical resection of specimens or percutaneous biopsy: because the pathological classification of lung adenocarcinoma would have been influenced by the subjective experience of the pathologists, the pathological classification was confirmed by two pathologists who had worked for 10 years after reaching a consensus according to the new 2015 classification criteria for lung adenocarcinoma; and (v) treatment-naive cases before HRCT. Cases that did not meet the diagnostic requirements where only a routine CT scan was performed or the respiratory motion artifact was too severe were excluded.

### 2.2 Examination Methods

A Brilliance 64-slice CT (Philips Medical Systems, Netherlands) machine was used for scanning. All patients received strict breathing training before scanning, adopted the head-first supine position, and adopted an end-inspiratory hold during scanning. The scanning scope covered all areas from the apices to the bottom of the lungs. Exposure conditions: 120 kV, 150 mA, collimation 0.625 mm × 64, pitch 0.64, scanning and reconstruction matrix both at 1024 × 1024, reconstruction slice thickness and interval both at 0.67 mm. Scanning image observation: mediastinal window (window position: 30–50 Hu; window width: 250–350 Hu); pulmonary window (window position: −450 to −600 Hu; window width: 1500–2000 Hu). The conventional CT scan images could not accurately identify lung GGNs; therefore, this study did not use them.

### 2.3 Image Analysis

**Figure 1:**
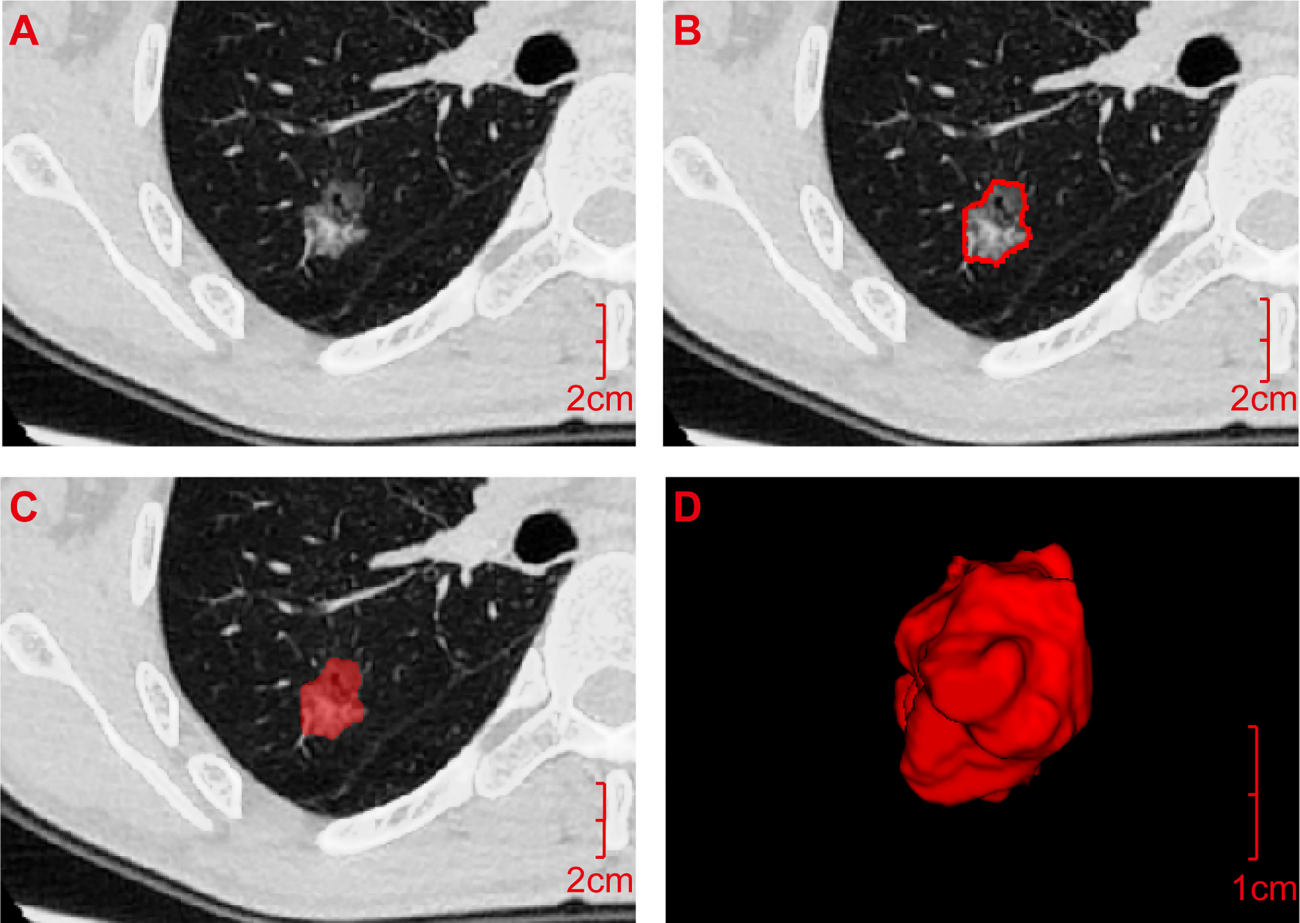
Acquisition process for the radiomics features. **(A)** Digital Imaging and Communications in Medicine image of the transverse section of the ground-glass nodule was pre-processed by A.K. software and then imported into ITK-SNAP software. **(B)** ROI was manually outlined layer by layer along the inner edge of the lesion based on pixel points. **(C)** ROI was outlined. **(D)** It was fused and saved as an ROI three-dimensional image and imported into A.K. software together with the pre-processed original image **(A)** in batches to quantitatively calculate the radiomics features. ROI, Region of interest.

First, all Digital Imaging and Communications in Medicine images of lung GGNs were imported into the A.K. (Analysis Kit) software developed by GE (USA) for pre-processing. Then, ITK-SNAP software (Version 3.6) was used to manually outline the regions of interest (ROIs) layer by layer along the inner edges of lung GGNs based on pixel points, and then they were fused and saved into three-dimensional (3D) images (**Figure 1**). All lung GGN images were outlined by a resident who had worked for 5 years and a deputy chief physician who had worked for 15 years. The intraclass correlation coefficient (ICC) was used for consistency analysis, and an ICC >0.8 indicated good consistency (22). The sketchers were blinded to the pathological results of the lung GGNs.

To acquire radiomics feature, the original images of lung GGNs after pre-processing and the corresponding ROI 3D images were imported into A.K. software in batches, and six types of radiomics features were quantitatively calculated: histogram, form factor, texture, gray level co-occurrence matrix (GLCM), run-length matrix (RLM), and gray level zone size matrix (GLSZM).

### 2.4 Statistical Methods

#### 2.4.1 Statistical Analysis of the Clinical Data

Using R language software (Version 3.5.1), descriptive statistical analysis was carried out between the training and verification groups. The chi-square test was used for qualitative variables, and the t-test or rank sum test was used for continuous variables, with P<0.05 indicating that the difference was statistically significant. In addition, the Bootstrap method is used to estimate the confidence interval.

#### 2.4.2 Screening of Radiomics Features and Construction of a Random Forest Prediction Model

In this study, random forest combined with hyperparameter tuning was used for prediction modeling. As a leader of ensemble learning methods, random forest trains decision tree models with partial data and partial features and then fuses these tree models, and finally, uses voting to solve classification problems. Random forest can directly deal with high-dimensional data, and there is no need for feature screening before modeling. Random forest hyperparameter tuning includes model framework parameters and decision tree parameters. For model framework parameters, the number of weak learners is mainly tuned, i.e., the number of decision trees, and the range set in this study was 50–500. Decision tree parameters tuning includes tree depth (3–10 layers) and the number of leaf nodes (10–50). Using random grid search and 10-fold cross-validation, the results of hyperparameter tuning in each iteration were evaluated by accuracy. Finally, we found the best random forest hyperparameters. Based on the training data, we found the following optimal hyperparameters in this study: the number of decision trees was 184, the tree depth was 5, and the number of leaf nodes was 20. The best random forest model was also utilized in the training data to assess the importance of features and feature selection.

Using this set of hyperparameter settings, prediction analysis of the training group and verification group based on the random forest algorithm was carried out again, and the receiver operating characteristic (ROC) curve was used to evaluate the prediction efficacy of the model. The calibration curve was used to evaluate the consistency between the probability predicted by the model and the actual probability.

## 3 Results

### 3.1 Comparison of the General Clinical Data of Patients in the Training and Verification Group

**Figure 2:**
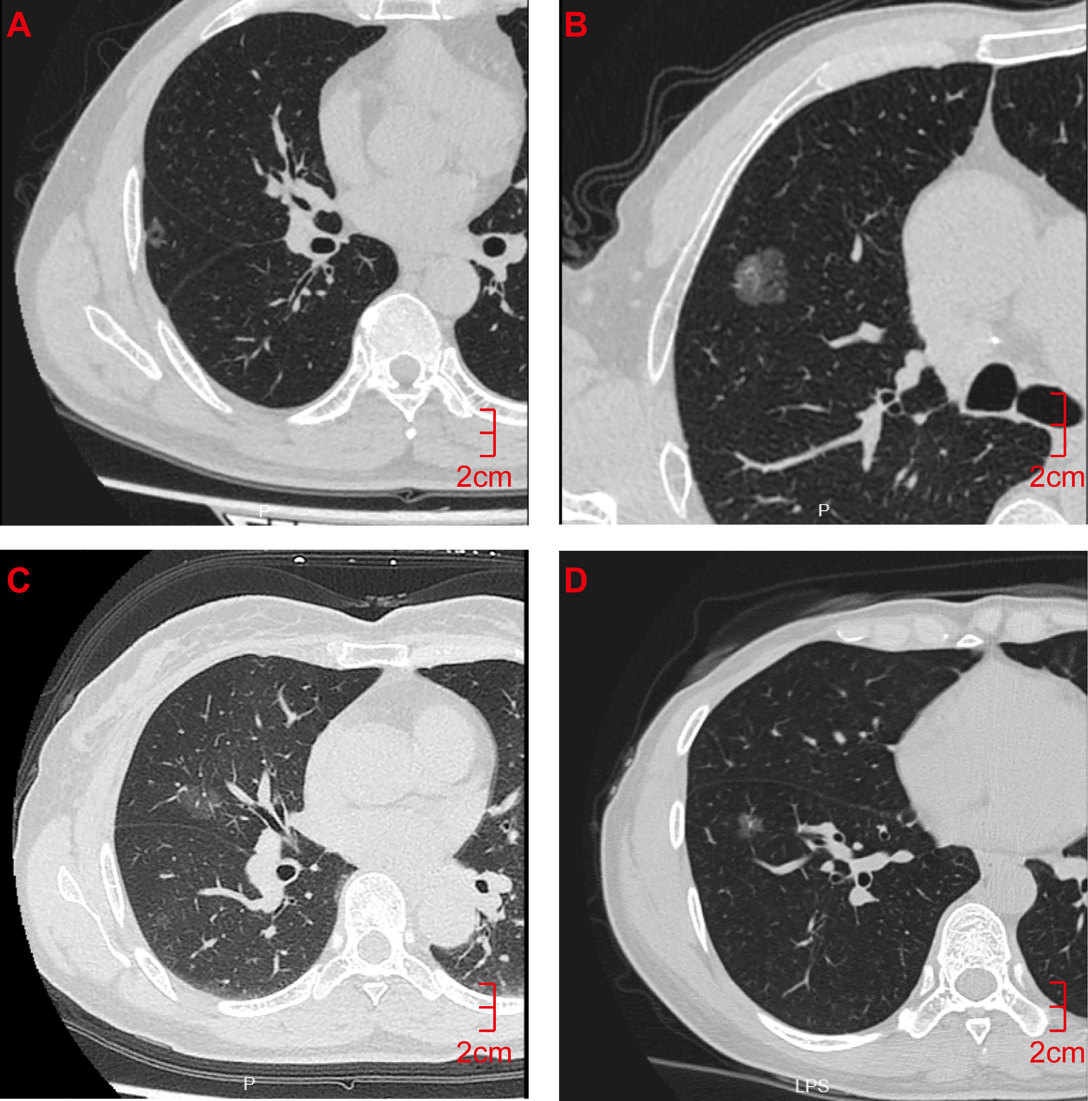
CT Findings of representative IAC and MIA nodules. **(A)** M/55y, pGGN of the right upper lobe with a long diameter of 1.2 cm, pathological diagnosis showed that the lesion was IAC. **(B)** M/32y, mGGN of the right upper lobe with a long diameter of 1.5 cm, pathological diagnosis showed that the lesion was IAC. **(C)** F/62y, pGGN with a long diameter of 1.2 cm in the right middle lobe, pathological diagnosis showed that the lesion was MIA. **(D)** F/55y, pGGN with a long diameter of 1.0 cm in the right lower lobe, pathological diagnosis showed that the lesion was MIA. CT, computed tomography; pGGN, pure ground-glass nodule; mGGN, mixed ground-glass nodules; IAC, invasive adenocarcinoma; MIA, minimally invasive adenocarcinoma.

A total of 118 lung GGNs [36 pure GGNs (pGGNs) and 82 mixed GGNs (mGGNs)] in 106 patients were included in this study, including 27 men (25.5%) and 79 women (74.5%) whose ages ranged from 28 to 76 years, with an average age of 55.61 ± 11.50 years. The surgical and pathological analysis confirmed 61 IAC lesions in 56 patients and 57 MIA lesions in 53 patients; 42 lesions were located in the left lung (18 in the upper left lobe and 24 in the lower left lobe), and 76 in the right lung (10 in the upper right lobe, 42 in the middle right lobe, and 24 in the lower right lobe) (**Figure 2**). Thirteen patients had a history of smoking, and eight had a history of a malignant tumor, all of which were lung cancer, two of them also had a history of thyroid cancer. Ninety-six patients had single lung GGNs, whereas 10 had multiple, among which two patients had three lung GGNs resected at the same time. Postoperative pathology results showed three IAC lesions in one patient and two IAC lesions and one MIA lesion in the other patient. All patients underwent video-assisted thoracoscopic surgery, 56 patients underwent lobectomy, 40 patients underwent sublobar resection (segmental resection or wedge resection), and 10 patients underwent lobectomy and sublobar resection. No lymph node or distant metastasis was found in any patient after the operation. The random function of R language software (Version 3.5.1) was used to divide the 118 lung GGNs into the training group and verification group at a ratio of 7:3. There were 83 lesions (including 43 IAC lesions and 40 MIA lesions) in the training group and 35 lesions (including 18 IAC lesions and 17 MIA lesions) in the verification group. There was no significant difference between IAC and MIA in terms of age, gender, smoking history, tumor history, and lung GGN location in both groups (P>0.05) (**Table 1**).

**Table 1:** Comparison of the general clinical data of patients in the training and verification groups (N = 118).

Clinical data	Training group			Verification group		
	IAC	MIA	*P*^1^	IAC	MIA	P
Number of cases (cases)	42	38		16	15	
Number of lesions (number)	43	40		18	17	
Age (years)	56.21 ± 11.15	54.23 ± 9.98	0.3978	56.94 ± 12.80	54.71 ± 11.62	0.5938
Gender (male/female)	(12/30)	(9/29)	0.6198	(5/11)	(4/11)	0.2504
Smoking history (cases)	4	5	0.1109	2	2	0.6406
Tumor history (cases)	3	2	0.2476	1	2	0.0629
GGN location (left/right)	(16/27)	(12/28)	0.4876	(9/9)	(5/12)	0.2140

P<0.05 indicates statistically significant difference.

GGN, ground-glass nodule; IAC, invasive adenocarcinoma; MIA, minimally invasive adenocarcinoma.

### 3.2 Acquisition and Screening of Radiomics Features

**Figure 3:**
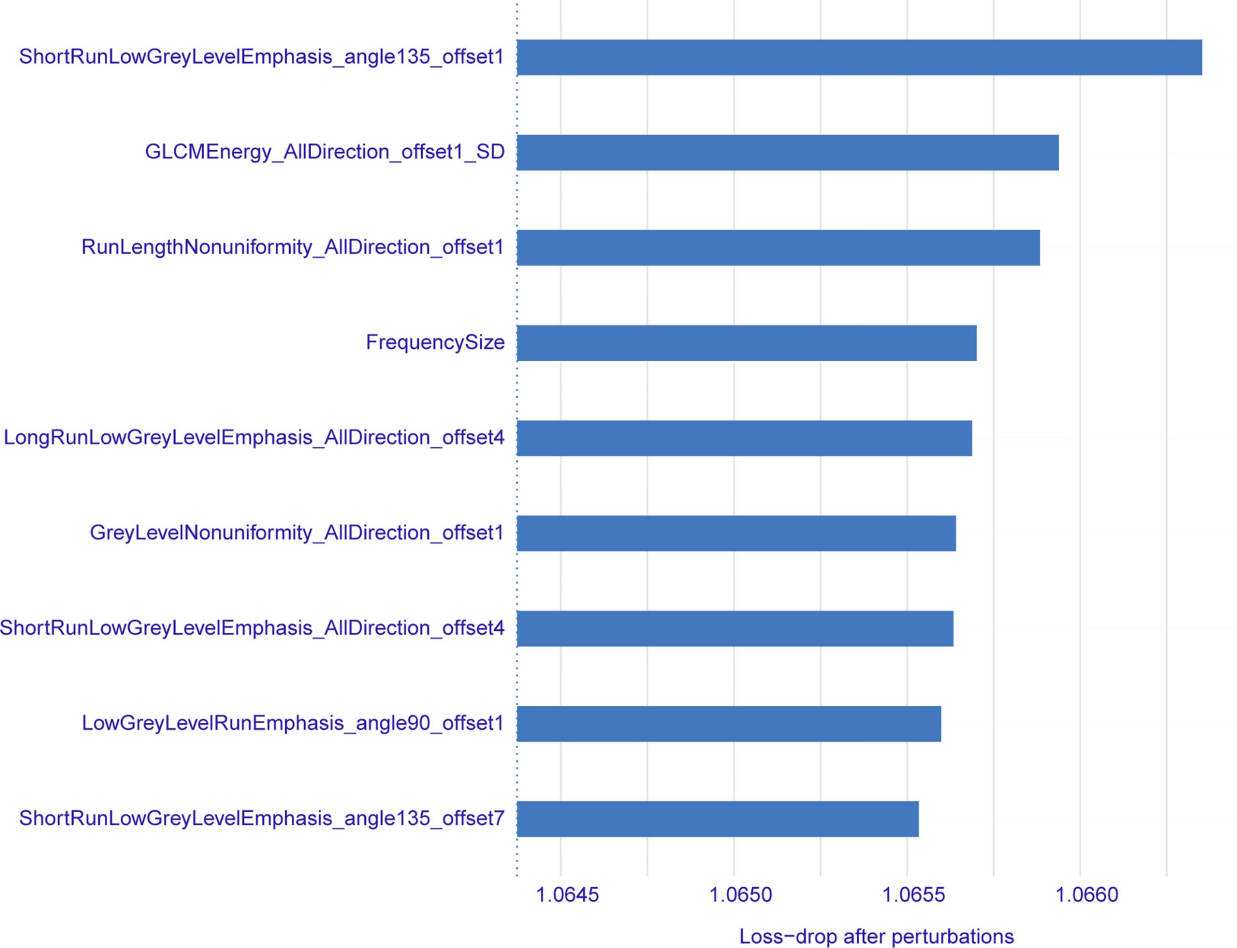
Names, importance, and sorting of radiomics features.

**Figure 4:**
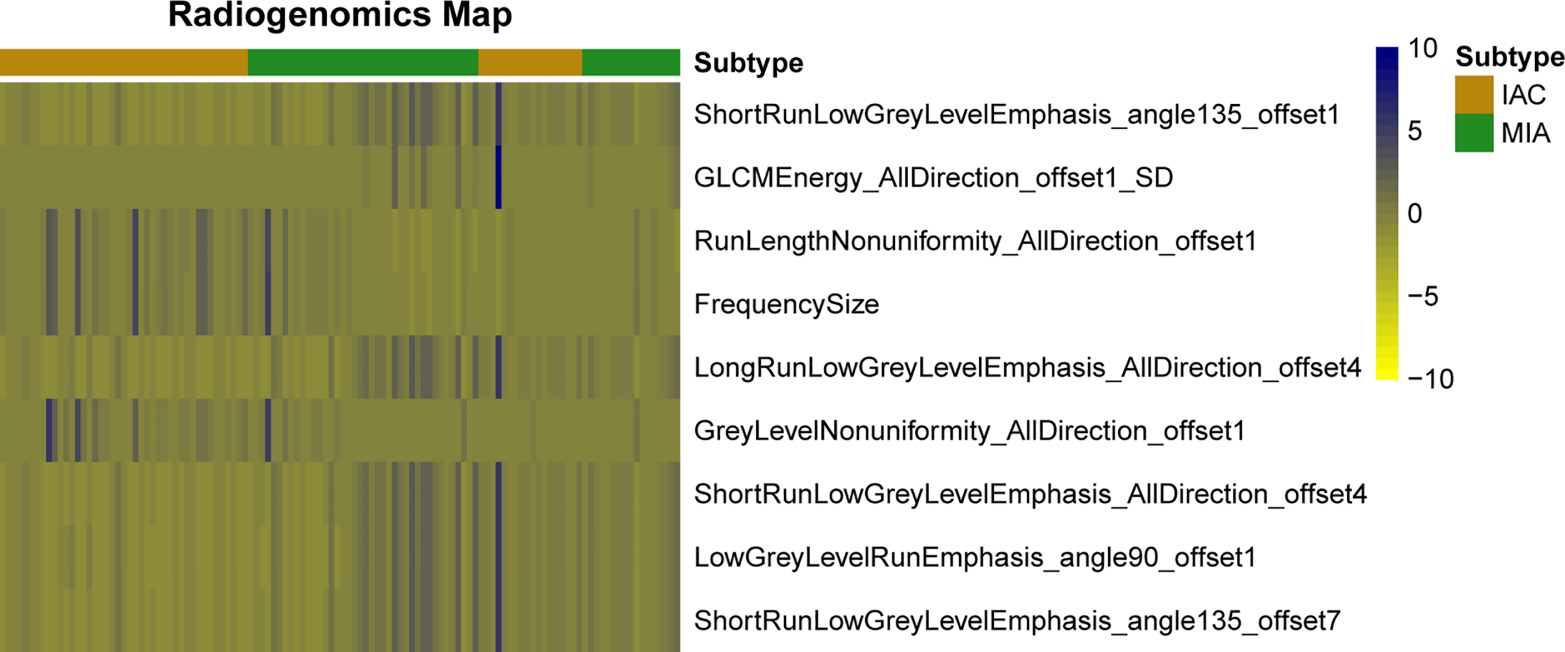
Heat map of radiomics features. Each row represents a feature, and each column represents a lung ground-glass nodule. The figure shows the difference between invasive adenocarcinoma and minimal invasive adenocarcinoma in each feature and indicates the classification ability of the features.

A total of 396 valid radiomics features in six categories were collected by A.K. software for each lung GGN (**Table 2**): 42 histogram, 9 form factor, 144 texture, 11 GLCM, 180 RLM, and 10 GLSZM features. The ICC was used for consistency analysis, and the characteristic features with an ICC <0.8 were eliminated. Based on the data of the training group, random forest combined with hyperparameter tuning was used to tune parameters and evaluate the importance of the radiomics features (19). Then we extract the top-n features for training and evaluation, and the experiment reveals that the top-9 features yields the best outcome. Finally, nine optimal radiomics features were screened out. The names, categories, importance, and ICC of each radiomics features were shown in **Table 3** and **Figure 3**, and the difference between IAC and MIA in each feature was statistically significant (*P*<0.05) (**Table 4**). A total of nine features were classified into three main categories: RLM, gray level co-occurrence matrix, and histogram features. Among them, the seven RLM features included three short run low grey level emphasis features, one long run low grey level emphasis feature, one run length nonuniformity feature, one grey level nonuniformity feature, and one low grey level run emphasis feature. There was one gray level co-occurrence matrix feature and one histogram feature, which were GLCM energy and frequency size, respectively. **Figure 4** shows the distribution of the values of the radiomics features of all the IAC and MIA patients mentioned above in the training and test datasets.

**Table 2:** Types and numbers of valid radiomics features.

Type of valid radiomics features	n
Histogram features	42
Form factor features	9
Texture features	144
Gray level co-occurrence matrix features	11
Run-length matrix features	180
Gray level zone size matrix features	10

**Table 3:** Names, categories, importance, and ICC of the selected radiomics features.

Radiomics features	Importance	ICC
**Run-length matrix features**		
Short run low grey level emphasis_angle135_offset1	1.06635	0.988
Run length nonuniformity_AllDirection_offset1	1.06588	0.997
Long run low grey level emphasis_AllDirection_offset4	1.06569	0.993
Grey level nonuniformity_AllDirection_offset1	1.06564	0.984
Short run low grey level emphasis_AllDirection_offset4	1.06563	0.995
Low grey level run emphasis_angle90_offset1	1.06560	0.982
Short run low grey level emphasis_angle135_offset7	1.06554	0.965
**Gray level co-occurrence matrix features**		
GLCM energy_AllDirection_offset1_SD	1.06594	0.999
**Histogram features**		
Frequency size	1.06570	0.996

GLCM, gray level co-occurrence matrix; SD, square deviation; ICC, intraclass correlation coefficient.

**Table 4:** Comparative analysis of selected radiomics features between IAC and MIA.

Radiomics Features	Pathological type	Value	*P*
Short run low grey level emphasis_angle135_offset1	IAC	4.24×10^-4^ ± 4.31×10^-4^	0.0000
	MIA	1.55×10^-3^ ± 1.13×10^-3^	
Run length nonuniformity_AllDirection_offset1	IAC	1705.55 ± 1686.94	0.0013
	MIA	320.71 ± 380.09	
Long run low grey level emphasis_AllDirection_offset4	IAC	5.81×10^-3^ ± 6.97×10^-3^	0.0005
	MIA	2.39×10^-2^ ± 1.80×10^-2^	
Grey level nonuniformity_AllDirection_offset1	IAC	19.56 ± 24.42	0.0000
	MIA	4.72 ± 5.61	
Short run low grey level emphasis_AllDirection_offset4	IAC	4.20×10^-4^ ± 4.27×10^-4^	0.0000
	MIA	1.54×10^-3^ ± 1.12×10^-3^	
Low grey level run emphasis_angle90_offset1	IAC	1.50×10^-3^ ± 1.76×10^-3^	0.0000
	MIA	6.05×10^-3^ ± 4.51×10^-3^	
Short run low grey level emphasis_angle135_offset7	IAC	4.20×10^-4^ ± 4.26×10^-4^	0.0000
	MIA	1.53×10^-3^ ± 1.12×10^-3^	
GLCM energy_AllDirection_offset1_SD	IAC	1.21×10^-8^ ± 4.18×10^-8^	0.0000
	MIA	5.93×10^-7^ ± 2.13×10^-6^	
Frequency size	IAC	1821.46 ± 1838.75	0.0000
	MIA	340.68 ± 417.84	

P<0.05 indicates statistically significant difference.

IAC, invasive adenocarcinoma; MIA, minimally invasive adenocarcinoma; GLCM, gray level co-occurrence matrix; SD, square deviation.

### 3.3 Prediction Efficacy of the Radiomics Random Forest Model

**Figure 5:**
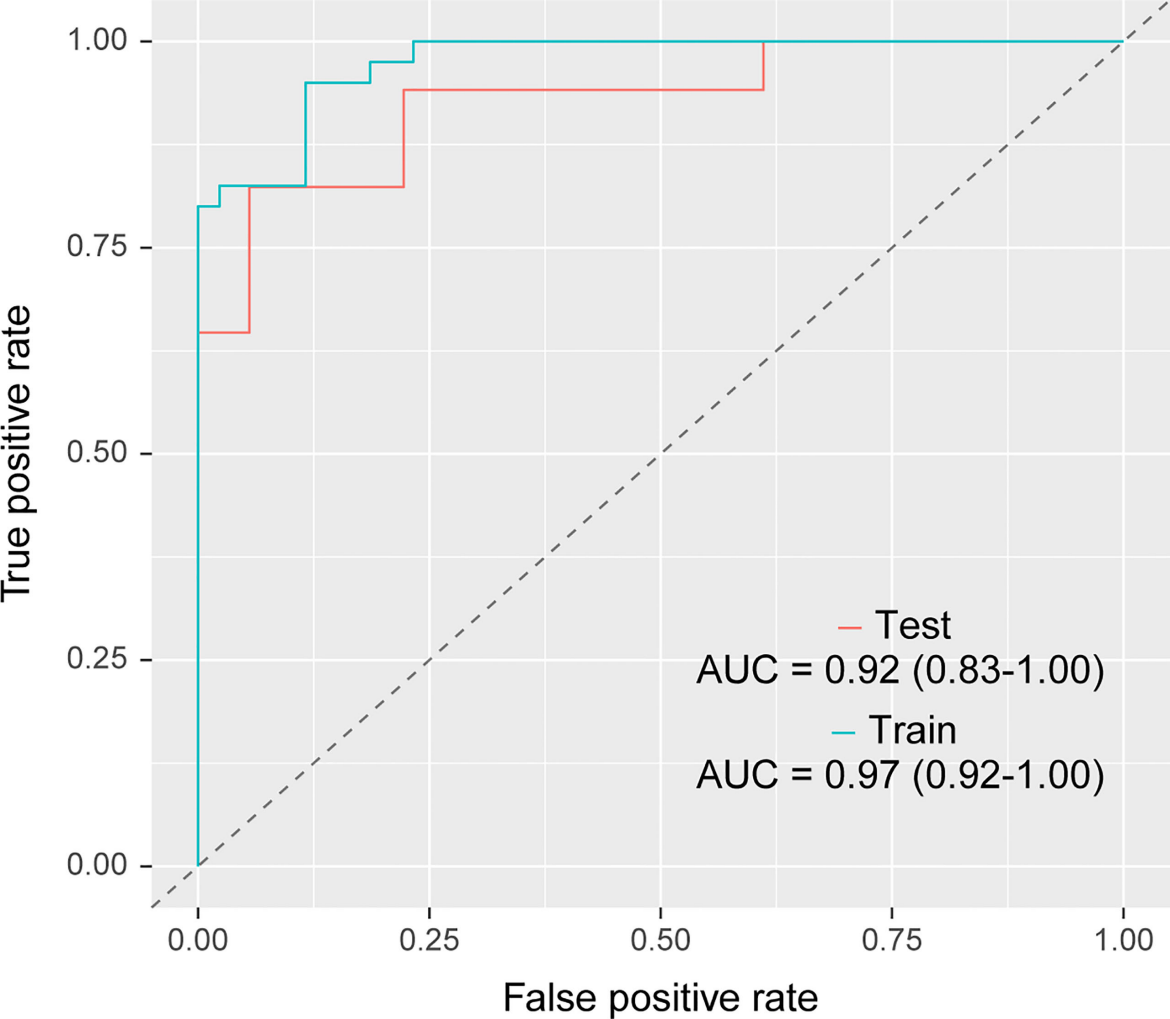
Receiver operating characteristic curve analysis of the radiomics random forest prediction model in differentiating lung ground-glass nodule-type invasive adenocarcinoma and minimal invasive adenocarcinoma in the training group (blue line) and the verification group (red line). The areas under the curve of the training and verification groups were 0.97(95%CI, 0.92-1.00) and 0.92 (95%CI, 0.83 - 1.00), respectively. CI, confidence intervals.

**Figure 6:**
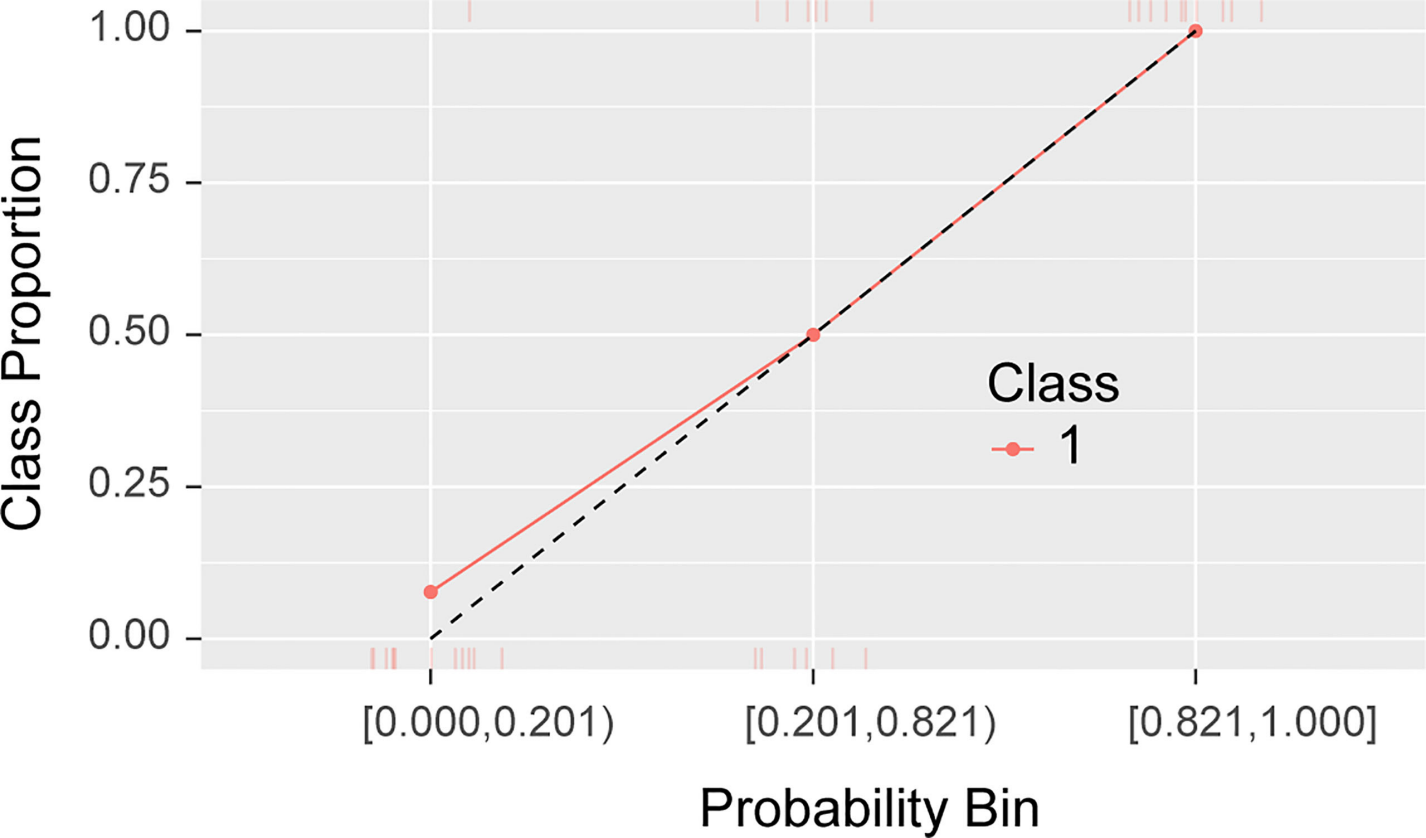
Calibration curve of the radiomics random forest prediction mode.

Based on the data of the training group, random forest combined with hyperparameter tuning results were used for prediction modeling, and prediction analysis of the training group and verification group based on the random forest algorithm was carried out again with this set of hyperparameter settings; the ROC curve was used to evaluate the prediction efficacy of the model (**Figure 5**). The accuracy for the training group was 0.89 (95%CI, 0.73 - 0.99), sensitivity was 0.98 (95%CI 0.78 - 1.00), specificity was 0.81 (95%CI, 0.59 - 1.00), and area under the curve (AUC) was 0.97(95%CI, 0.92-1.00); the accuracy for the verification group was 0.80 (95%CI, 0.58 - 0.93), sensitivity was 0.82 (95%CI, 0.55 - 1.00), specificity was 0.78 (95%CI, 0.57 - 1.00), and AUC was 0.92 (95%CI, 0.83 - 1.00). The calibration curve of the prediction model (**Figure 6**) showed good agreement between the predicted probability and actual probability.

## 4 Discussion

### 4.1 Epidemiology

Lung cancer is the leading cause of cancer-related death worldwide (23) and at present, it has the fastest growing prevalence and mortality among human malignancies (24) and the trend is rising. Adenocarcinoma is the most common pathological type of lung cancer, accounting for approximately 50% of cases of lung cancer. The proportion of males is higher than that of females, and smoking is the most important risk factor (25). In this study, the proportion of female patients was much higher than that of male patients (79/27), and the proportion of smokers (13/106) was relatively low, which is inconsistent with previous reports. It might be related to the case selection in this study, and at the same time, it is necessary to consider that the incidence in females has been increasing year after year due to indoor air pollution and second-hand smoke exposure, among other factors. There were no significant differences in age, gender, smoking history, tumor history, or GGN location between IAC and MIA patients, which is consistent with the literature (26). The National Lung Screening Trial in the United States showed that CT low-dose screening was helpful in reducing the mortality of lung cancer (27). In recent years, the detection rate of GGN lung adenocarcinoma has been increasing, and early diagnosis and accurate pathological classification have become the keys to treatment. However, due to the overlapping of traditional imaging manifestations among pathological subtypes, it is still difficult to accurately classify them, especially distinguishing between IAC and MIA, which are both invasive lesions. Therefore, a systematic and objective differential diagnosis method must be urgently developed.

### 4.2 Correlation Between Radiomics Features and Pathological Subtypes of GGN Lung Adenocarcinoma

Through univariate analysis, in the verification group, there was no significant difference in tumor history between IAC and MIA (P=0.0629). However, due to the small sample size, the results presented here should be carefully considered. Notably, since a purely radiomics prediction model was employed in this study, biological differences in tumor history have limited potential impact on model efficacy. When using combined clinical-radiomic prediction models, propensity score matching should be used to control for confounding variables in the presence of statistically significant biological differences in tumor history to improve data comparability. Using radiomics, numerous imaging features can be extracted *via* software analysis of lesion heterogeneity, which is objective, does not cost much and facilitates the prediction of clinical outcomes (13). In this study, nine optimal radiomics features were screened out by combining random forest with hyperparameter tuning, which were classified into three categories: RLM, gray level co-occurrence matrix, and histogram features. Except for one histogram feature that belongs to the low-order texture, the other eight features belong to the high-order texture which shows the distribution of pixel points. This also reveals that high-order texture can better reflect the spatial heterogeneity changes of lung GGNs.

The histogram is a function of the image gray level (28) which describes and compares the distributions of pathological or biological indicators quantitatively. Histogram features are related to the attributes of a single pixel, and the distribution of voxel intensity in CT images is described by common and basic indices; thus, the calculation results of voxel values are more accurate. It has been reported that the histogram pattern based on CT pixels can assist in distinguishing AAH from bronchioloalveolar carcinoma (29, 30). Although frequency size was the only histogram feature selected in this study, it ranked fourth in importance (**Figure 3**), which reflects its value in terms of the differential diagnosis of IAC and MIA. The GLCM is a two-dimensional gray histogram that examines a pair of pixels separated by a fixed spatial relationship, which reflects the change speed and amplitude of pixel gray levels at different intervals and in different directions in the image. It is the basis for analyzing the arrangement rules and local patterns of images, including 11 indices such as energy, entropy, inertia, and correlation (31), which reflect the internal characteristics and spatial heterogeneity of tumors. Energy is a set of feature values that indicates the complexity of image texture. A large energy value indicates that the image has very good uniformity or very similar pixels, and vice versa. Heterogeneity of malignant tumors is caused by tissue structure changes resulting from uneven distribution of cell density, hemorrhage, necrosis, and mucoid degeneration, among other factors (32). Pathologically, IAC is more heterogeneous than MIA, and the complexity of image texture is also higher, though it is not easy to detect using the naked eye. The energy value of IAC in this study was significantly less than that of MIA (**Table 4**), which objectively reflected the difference in image texture between them. The RLM features mainly reflect the roughness and directionality of the image. Directional texture will have a longer run length at a certain angle, in which the value of short run emphasis is larger on rougher images, and that of long run emphasis is larger on smoother images. The run length is related to the gray level distribution of the image, and the heterogeneity of the tumor often reflects the gray level changes of the image; thus, the RLM is very sensitive to the texture changes of lung GGNs. Among the nine optimal radiomics features that were finally selected, seven were RLM features, and short run emphasis ranked first in importance (**Figure 3**). This shows that the RLM is most valuable for distinguishing IAC from MIA.

Form factor features are of great value in the differential diagnosis of pathological subtypes of GGN lung adenocarcinoma. Chae et al. (33) showed that mass (volume*density) was significantly different between pre-invasive lesions (AAH/AIS) and invasive lesions (MIA/IAC) presenting as mGGNs. However, none of the form factor features in this study were selected as an optimal feature, which might be related to the similarity and overlapping of morphological features between IAC and MIA, which is why it is more difficult to differentiate them.

### 4.3 Value and Superiority of the Radiomics Random Forest Model

Quantitative features such as the geometry, wavelet, and texture in the internal space of tumors were collected in a high-throughput way using radiomics software. The characteristic variables were screened out by computer artificial intelligence technology, and a quantitative prediction model was constructed, which made it a brand-new imaging diagnosis decision-making and analysis tool. Its application scope covers the qualitative analysis, clinical staging and grading, treatment outcome evaluation, and prognosis prediction of tumors (34).

At present, studies using radiomics prediction models to distinguish pathological subtypes of lung adenocarcinoma mainly focus on MIA/IAC and AAH/AIS or IAC and AIS/MIA, and have achieved promising results. For example, the predictive model between IVA and AIS/MIA based on pGGNs established by Xu et al. (35), the AUC value of the combined model was 0.848 (95% CI, 0.750-0.946); the combined clinical model of Wu et al. (36) - The AUC values ​​of the radiological model were 0.917 and 0.876 in the training and validation groups, respectively. It is worth noting that in 2021, the “WHO Classification of Thoracic Tumors (5th Edition)” has excluded AIS from the category of lung malignancies and classified it as a glandular precursor lesion together with AAH. Although both MIA and IAC require surgical treatment, MIA is suitable for sublobar resection (wedge resection or segmentectomy) with a 5-year disease-free survival rate of approximately 100% after complete resection, and IAC is suitable for standard lobectomy and extensive lobectomy. With lymph node dissection, the 5-year disease-free survival rate was 74.6%. Therefore, accurate preoperative identification of IAC and MIA will help guide the selection of surgical methods and the judgment of prognosis. Currently, studies to differentiate MIA from IAC are very rare. A previous study (18) constructed a combined prediction model integrating lesion shape and radiological features to distinguish MIA from IAC, with an AUC of 0.888. In this study, by using the same equipment, using the same scanning protocol and the same reconstruction scheme to acquire images, and using AK software for image preprocessing to ensure image consistency, we finally obtained AUC superior to the above studies values.

Fan et al. (37) established an individualized prediction model based on the patient’s age, spicule sign, pleural indentation sign, and radiomic labels, and established a clinical model based on the patient’s age, spicule sign, and pleural indentation sign to distinguish GGN lung adenocarcinoma from invasive lesions (AAH/AIS). The results showed that the AUC increased from 0.743 in the clinical model to 0.934 in the individualized prediction model, indicating the importance of radiomic labeling. She et al. (38) included 402 patients with lung GGNs and extracted 60 radiomics features, among which five features were the most critical diagnostic factors. The results showed that the AUCs of the radiomics prediction model in the training group and verification group were 0.95 and 0.89, respectively, indicating that radiomics had advantages in differentiating IAC from MIA/AIS. Weng et al. (18) included 119 pulmonary mGGN patients to differentiate IAC from MIA and extracted 396 radiomics features, among which four were optimal distinguishing features for establishing a radiomics model. The results showed that the AUCs of the radiomics feature model for the training and verification groups were 0.854 and 0.813, respectively. Then, a CT feature model was established using lesion morphology and the diameter of the solid components; the AUC was 0.755. Finally, the lesion morphology and radiomics features were combined, and the AUC was 0.888. In this study, A.K. software was used to collect the radiomics features; the optimal radiomics features were screened out by random forest combined with hyperparameter tuning and a prediction model was established. The results showed that the AUCs of the training and verification groups were 0.97(95%CI, 0.92 - 1.00) and 0.92 (95%CI, 0.83 - 1.00), respectively, indicating that the prediction model could differentiate IAC and MIA presenting as lung GGN non-invasively. This study established a pure radiomics labeling model, and the differentiation objects were IAC and MIA with more similar pathological features. The prediction efficacy was similar to that of individualized prediction models, which might be related to the fact that this study collected cases scanned with the same CT machine and the same scanning protocol, and pre-processed all images before outlining ROIs, which reduced the influences of equipment and scanning parameters on the results to some extent.

Chae et al. (33) used an artificial neural network to establish a radiomics prediction model to distinguish pre-invasive lesions (AAH/AIS) from invasive lesions (MIA/IAC) of mGGN lung adenocarcinoma and achieved good results (AUC=0.981). Although an artificial neural network can solve dichotomy problems well, its generalization ability in specific models is limited due to potential over-fitting and a complex structure. Random forest is an algorithm that integrates multiple decision trees through the idea of ensemble learning, and it essentially belongs to the ensemble learning method in machine learning. It can handle both continuous data and discrete data and can also handle missing data and sort the importance of variables. The results of random forest models have higher accuracy and generalization performance; thus, they are often used to predict the risk of diseases and susceptibility of patients (39).

### 4.4 Limitations

This study had some limitations. First, owing to the retrospective nature of the study, there is potential bias; thus prospective studies are required to confirm our results. Second, the manual sketching of ROIs made it difficult to eliminate bronchi and blood vessels in the nodules; thus, the accuracy of some features might be affected. Third, the boundary between some nodules and normal lung tissue was unclear, and boundary leakage might occur during image segmentation. Moreover, similar to some previous studies (22, 35, 36, 40), this study divides the lesions are divided into train and test sets – this leads to the possibility of overfitting as two lesions from the same patient may end up in different subsets. Finally, the sample size was too small, and the prediction accuracy of the model might be unstable to some extent. Therefore, future studies with larger sample sizes are warranted.

## 5 Conclusion

The radiomics prediction model established by combining random forest with hyperparameter tuning could effectively differentiate IAC and MIA presenting as lung GGN and could provide a noninvasive, low-cost, rapid, and reproducible preoperative prediction method that is clinically applicable.

## Data Availability Statement

The original contributions presented in the study are included in the article/supplementary material. Further inquiries can be directed to the corresponding authors.

## Ethics Statement

The studies involving human participants were reviewed and approved by Dongyang Hospital ethics review board. The patients/participants provided their written informed consent to participate in this study.

## Author Contributions

Z-HH and X-MY designed the study. F-HZ and H-JF collected and analyzed the relevant literature. K-FS, LZ, Z-ZP, C-LF, Z-BY, M-KW, and J-HS analyzed the literature and constructed the figures. All authors participated in writing the manuscript and have approved the final version of the manuscript for submission.

## Conflict of Interest

The authors declare that the research was conducted in the absence of any commercial or financial relationships that could be construed as a potential conflict of interest.

## Publisher’s Note

All claims expressed in this article are solely those of the authors and do not necessarily represent those of their affiliated organizations, or those of the publisher, the editors and the reviewers. Any product that may be evaluated in this article, or claim that may be made by its manufacturer, is not guaranteed or endorsed by the publisher.
